# Supplementary material for: NFκB-Mediated Mechanisms Drive PEDF Expression and Function in Pre- and Post-Menopausal Oestrogen Levels in Breast Cancer
Source: Int J Mol Sci. 2022 Dec 9;23(24):15641. doi: 10.3390/ijms232415641 (PMC9779285; doi:10.3390/ijms232415641)
Supplement: Supplementary file 1 [file ijms-23-15641-s001.zip › ijms-2042920-supplementary.pdf]

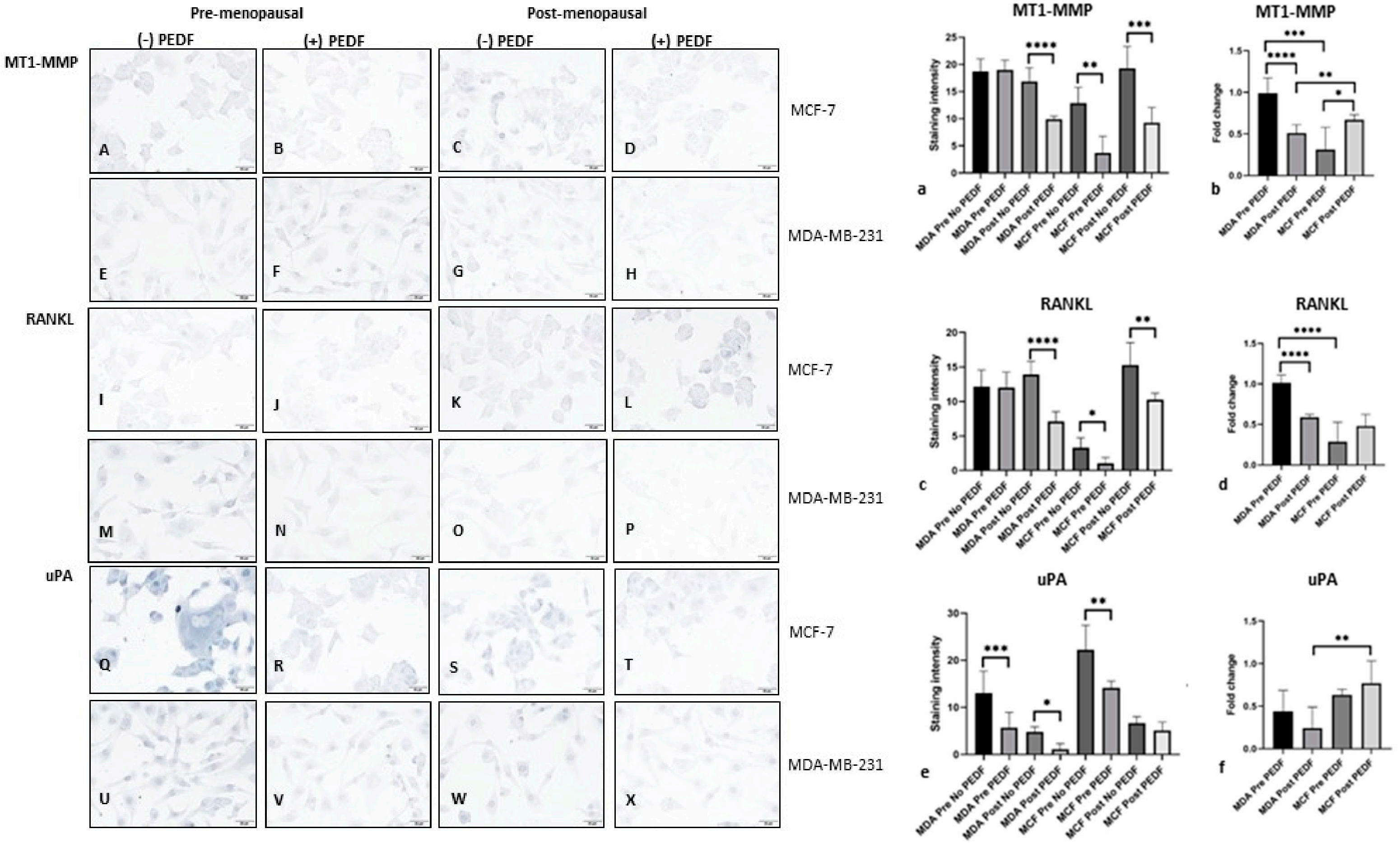

Supplementary Figure S1. Biomarker immunocytochemistry in MCF-7 and MDA-MB-231 cells following PEDF treatment in pre- versus post-menopausal oestrogens. Biomarker expression determined via immunocytochemistry (ICC) staining (blue) in MCF-7 (A-D, I-L, Q-T) and MDA-MB-231 (E-H, M-P, U-X) cells following incubation in media supplemented with either pre-menopausal oestrogens or post-menopausal oestrogens and treated with recombinant PEDF (100nM) or control for 24-hours. MT1-MMP ICC staining under pre-menopausal conditions in MCF-7 treated with control (A) or PEDF (B) and in MDA-MB-231 treated with control (E) or PEDF (F), or under post-menopausal conditions in MCF-7 treated with control (C) or PEDF (D) and in MDA-MB-231 treated with control (G) or PEDF (H). RANKL ICC staining under pre-menopausal

conditions in MCF-7 treated with control (I) or PEDF (J) and in MDA-MB-231 treated with control (M) or PEDF (N), or under post-menopausal conditions in MCF-7 treated with control (K) or PEDF (L) and in MDA-MB-231 treated with control (O) or PEDF (P). uPA ICC staining under pre-menopausal conditions in MCF-7 treated with control (Q) or PEDF (R) and in MDA-MB-231 treated with control (U) or PEDF (V), or under post-menopausal conditions in MCF-7 treated with control (S) or PEDF (T) and in MDA-MB-231 treated with control (W) or PEDF (X). Bar graphs showing biomarker ICC staining intensity in cells treated with control versus PEDF for MT1-MMP (a), RANKL (c), uPA (d), and uPAR (e). Bar graphs showing fold change in PEDF-treated cells versus control in pre- or post-menopausal conditions for MT1-MMP (b), RANKL (d), uPA (f). *Scale bar* 50µM. * $p < 0.05$; ** $p < 0.01$; *** $p < 0.001$; **** $p < 0.0001$.

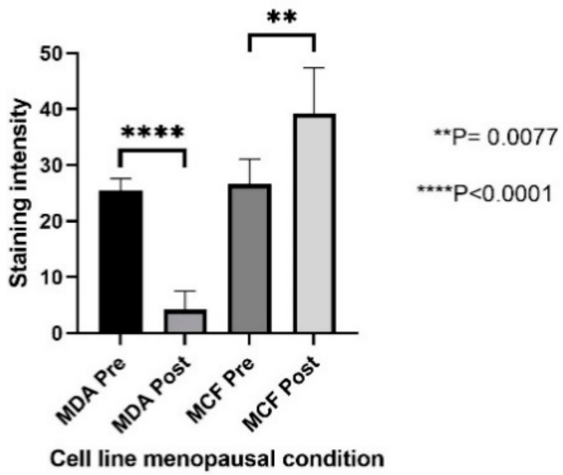

Supplementary Figure S2. p-NFκB-p65 expression in pre- versus post-menopausal oestrogens in MCF-7 and MDA-MB-231 cells. Phosphorylated (p-)NFκB-p65 expression determined via immunocytochemistry staining in MCF-7 and MDA-MB-231 cells following incubation in media supplemented with either pre-menopausal oestrogens or post-menopausal oestrogens for 24-hours.
